# Array-Based Whole-Genome Survey of Dog Saliva DNA Yields High Quality SNP Data

**DOI:** 10.1371/journal.pone.0010809

**Published:** 2010-05-25

**Authors:** Jennifer S. Yokoyama, Carolyn A. Erdman, Steven P. Hamilton

**Affiliations:** Department of Psychiatry and Institute for Human Genetics, University of California San Francisco, San Francisco, California, United States of America; Institut de Genetique et Microbiologie, France

## Abstract

**Background:**

Genome-wide association scans for genetic loci underlying both Mendelian and complex traits are increasingly common in canine genetics research. However, the demand for high-quality DNA for use on such platforms creates challenges for traditional blood sample ascertainment. Though the use of saliva as a means of collecting DNA is common in human studies, alternate means of DNA collection for canine research have instead been limited to buccal swabs, from which dog DNA is of insufficient quality and yield for use on most high-throughput array-based systems. We thus investigated an animal-based saliva collection method for ease of use and quality of DNA obtained and tested the performance of saliva-extracted canine DNA on genome-wide genotyping arrays.

**Methodology/Principal Findings:**

Overall, we found that saliva sample collection using this method was efficient. Extractions yielded high concentrations (∼125 ng/ul) of high-quality DNA that performed equally well as blood-extracted DNA on the Illumina Infinium canine genotyping platform, with average call rates >99%. Concordance rates between genotype calls of saliva- versus blood-extracted DNA samples from the same individual were also >99%. Additionally, *in silico* calling of copy number variants was successfully performed and verified by PCR.

**Conclusions/Significance:**

Our findings validate the use of saliva-obtained samples for genome-wide association studies in canines, highlighting an alternative means of collecting samples in a convenient and non-invasive manner.

## Introduction

Assemblies of the *Canis familiaris* genome [1], [2] have facilitated genomic research in the domestic dog, fostering discovery of genetic loci influencing a range of canine traits and diseases. Though targeted gene mapping efforts using microsatellite markers and resequencing of candidate genes have resulted in discoveries for traits with simple hereditary patterns, the study of complex disease and behavioral phenotypes has proven to be very challenging. However, with over 2.5 million single-nucleotide polymorphisms (SNPs) annotated on the canine genome, the potential for performing unbiased surveys for genetic loci underlying traits via genome-wide association study (GWAS) has become a practical tool for canine geneticists, leading to compelling association signals for traits with reduced genetic complexity [3]–[9]. Even a GWAS performed for presumably more complex phenotypes such as canine compulsive disorder (similar to human obsessive-compulsive disorder) has rendered promising results in a single genomic region [10]. Array-based genotyping platforms are now available and provide data for tens- to hundreds- of thousands of SNPs across the dog genome in a single genotyping assay.

Because array-based genome-wide genotyping platforms require large quantities of high quality genomic starting material, DNA for such studies has traditionally been obtained from whole blood. However, with increasing demands for large sample sizes to ensure statistical power to detect multiple signals of modest effect as is expected for complex phenotypes, obtaining whole blood samples from large numbers of dogs becomes challenging. In fact, sampling can even become the limiting factor when studying behavioral traits such as severe anxiety disorders where handling by a clinician in itself causes great duress to the animal, and is often only possible with sedation.

The utility of dog DNA obtained from buccal sampling is well established for microsatellite marker typing, targeted SNP genotyping and limited resequencing. We have found that use of whole-genome amplification (WGA) provided sufficient quantities of genomic material for use in higher throughput multiplex genotyping assays surveying up to several hundred SNPs [11]. Although WGA of canine buccal DNA produces reasonable (∼3 µg) quantities of total DNA [12], previous studies by our group suggest that only 3–15% (90–450 ng) of this total WGA sample actually represents canine DNA [11]. Use of WGA buccal DNA from dogs on genome wide arrays—which require 250–500 ng of genomic DNA input—is thus questionable, given the level of microbial DNA contamination. Preliminary studies by other groups have found performance of buccal swab DNA on Illumina's Infinium canine array to be modest, suggesting the total amount of canine DNA present in WGA buccal samples is insufficient for high-quality data production for use in GWAS (MW Neff, personal communication).

Another mode of DNA sampling that has gained increasing utilization is saliva collection, from which DNA has been shown to be of equivalent quality as blood-extracted DNA [13]. The most notable strengths of saliva collection involve convenience: 1) samples can be collected at home by users themselves; 2) once saliva is mixed with stabilization buffer samples are stable for several months at room temperature; and 3) saliva can be sent through postal mail and across international borders without infringement of shipping laws or ethical restrictions. Saliva collection has a higher return rate than blood in human subjects [13], [14]. Additionally, bacterial DNA content has been reported to compose only 16.1% of the total DNA obtained from canine saliva samples [15]. Perhaps most importantly, saliva collection provides a painless, non-invasive alternative to venous draws—one of the main reasons many researchers have switched to saliva collection for research in infants and children.

Saliva-extracted DNA has been demonstrated to be of equivalent quality as blood-extracted DNA in humans [13]. Very recently, Mitsouras and Faulhaber (2009) also demonstrated high yields of high quality DNA from canine saliva, sufficient for PCR-RFLP genotyping. We therefore proposed saliva collection as an alternative to blood draws for obtaining DNA samples from dogs in a minimally invasive fashion for use on genome-wide genotyping platforms to yield high-quality data for use in GWAS. We describe here our verification of DNA yield and quality, genotyping performance, copy number variant (CNV) calling, and data quality via comparison with blood-extracted DNA samples. We also report owner feedback from kit usage and highlight the utility of saliva collection for future studies in canine genetics.

## Materials and Methods

### Samples

Saliva and blood samples were collected from four Bearded Collies and one Border Collie in the context of our ongoing genetic studies of canine behavior. Saliva only was also obtained from six additional Bearded Collies recruited for the same study. Saliva samples were collected by owners using the Oragene·ANIMAL (OA-400 Tube Format, DNA Genotek, Ontario Canada) kit as per manufacturer's instructions. Briefly, saliva was collected from dog's mouth using 2–3 absorbent sponges (http://www.dnagenotek.com/DNA_Genotek_Support_Lit_UI_ANIMAL.html). After sample collection, DNA was preserved by placing the sponges in Oragene·ANIMAL stabilization solution, labeled, and then sent to our laboratory by mail. All saliva samples were stored at room temperature before and after shipping. Blood samples were obtained by 3–5 cc blood draw. All animal work was approved by the local review committee.

### DNA extraction

Extraction of dog DNA was performed as suggested by manufacturer's instructions except as noted based on our extensive experience with human saliva DNA. Samples were incubated for two hours in water at 50°C. Swabs absorbed the full volume of stabilization buffer in addition to saliva, and thus required manual extraction (‘squeezing’ with sterile tweezers) to remove solution for use in extraction. The solution was collected in original holding container, and then 500 µl was aliquoted via pipette into a 1.5 mL microcentrifuge tube. Absorbent sponges were kept in remaining stabilization solution in the event that additional extractions were required. Because twice the amount of solution was aliquoted for extractions, 20 µl of Purifier was used, and the use of glycogen was omitted. The Animal protocol contains a NaCl step to ensure efficient recovery of DNA; as this step is not in the Human protocol and was added between the two versions of the Animal protocol that we performed (beta testing kit courtesy of DNA Genotek vs. published version PD-PR-095 Issue 2.1), extractions were carried out both with and without the use of NaCl on the same sample (which was not used for the reported genotyping). The single Border Collie sample was extracted without the NaCl step via the beta kit instructions, whereas the ten Bearded Collie samples were all extracted using the NaCl step from the updated protocol. For the final hydration step, 100 µl of Hydration Buffer from the Qiagen kits used for blood extractions (Qiagen Inc., Valencia CA) was used to rehydrate DNA, and samples were incubated for at least 24 hours at room temperature prior to final storage at 4°C. Blood sample DNA was extracted in-house using standard methods with the Puregene Blood Kit (Qiagen Inc.).

### Quantification of DNA

Quantification of all extracted DNA samples was performed on a NanoDrop (ND-1000 v3.3.0) spectrophotometer (Thermo Fisher Scientific Inc., Wilmington DE). Quantification of saliva-extracted samples was not corrected as per notes suggested by DNA Genotek (Laboratory Protocol PD-PR-095 Issue 2.1), but rather were reported as calculated by the NanoDrop for direct comparison with results published by other groups for human saliva and blood. For more details, please see **Discussion**.

### Genotyping

Samples were genotyped on the Infinium Canine SNP20 BeadChip (Illumina Inc., San Diego CA) by the Genomics Core Facility at the University of California, San Francisco. Genotypes were called and quality control was conducted in-house using the GenomeStudio Data Analysis Software package (1.0.2.20706, Illumina Inc.). Clusters of all samples with GenTrain Scores (a measure of reliable SNP detection)<0.60 were visually assessed for quality and either manually reclustered or zeroed due to poor performance (i.e., excluded from the data set). Further exclusion criteria removed SNPs with call rates<95% or minor allele frequency (MAF)<0.02.

### CNVs

Copy number variation was evaluated *in silico* with the GenomeStudio software (cnvPartition v2.4.4, Illumina Inc.) using default criteria. One predicted CNV locus was also evaluated by direct PCR of two genomic segments within the putative deletion region using the following primer pairs: (PLSCR1exon amplicon) forward 5′-TCTAAACCCAGGATTAGCAAGAA-3′, reverse 5′-CCATGTAATTTTGATAGGGTATTTCA-3′ and (CFA23CNV44Mb amplicon) forward 5′-TGTAAACCTCATTTCACTTACATGG-3′, reverse 5′-GGTCCATGGAGGACTCTCTCT-3′. Platinum-Taq was used to amplify segments with a 58°C touchdown protocol in presence of 0.4 µM primer, 100 µM dNTPs, 2.5 mM Mg and 1 mM Betaine.

### Ethics Statement

All animal work was approved by Institutional Animal Care and Use Program at the University of California, San Francisco (AN079848-02). All dogs were recruited from private owners, who consented to use of de-identified data for research purposes.

## Results

### Saliva sample collection

Twelve sample kits were sent to six Bearded Collie owners who previously consented via written communication to participate in the sample collection. Of those, four owners representing 10 kits (dogs) returned samples to our laboratory, representing a 67% by-owner and 83% by-dog return rate. Surveys sent out with the beta testing version of the Oragene·ANIMAL saliva collection kits reported that owners found the collection to be very easy overall. For all owners, sample collection was successful and took less than 10 minutes.

### DNA yield

DNA extraction was successful for all saliva samples received from owners. For each sample, 500 µl of saliva-buffer solution was easily extractable from the swabs, with additional volume remaining after the liquid transfer step to the 1.5 mL tube, thus allowing for another extraction for more DNA if necessary. The extraction protocol was very straightforward, and DNA fibers were visible for all extractions performed. When quantified via NanoDrop spectrophotometer, saliva-extracted samples (n = 11) had a mean concentration of 125.5 ng/µl (Table 1), for an average yield of 12.6 µg of total DNA. This compares to the mean concentration of 384.4 ng/µl for our comparison blood-extracted samples (n = 5). The 260/280 mean for all saliva-extracted samples was 1.67, as compared to blood samples that had a mean of 1.96 (Table 1). However, the 260/230 mean for saliva-extracted samples was much lower than that of comparison blood samples, with an average of 0.53 for saliva versus 1.61 for blood (Table S1). Low 260/230 ratios suggest presence of contaminants, which absorb at 230 nm.

**Table 1:** Concentration, purity and concordance of saliva-versus blood-extracted DNA samples.

		Mean (range)		
Source	n	Concentration (ng/ul)	260/280	Concordance (%)
Saliva	11	125.5 (46.9–212.4)	1.67 (1.39–1.86)	–
Blood	5	384.4 (317–521.2)	1.96 (1.84–2.24)	*–*
Saliva vs. Blood	4	–	–	99.9 (99.9–100)

Mean values plus ranges for DNA concentration and 260/280 ratios (as a measure of purity) as calculated by NanoDrop spectrophotometer for saliva and blood samples, and mean genotype concordance for individuals represented by both saliva and blood. Concordance is the proportion of agreeing genotype calls over total genotypes that were called for both samples (saliva and blood). Source–tissue source of DNA extraction; n–number of dogs.

### Genotyping

**Figure 1:**
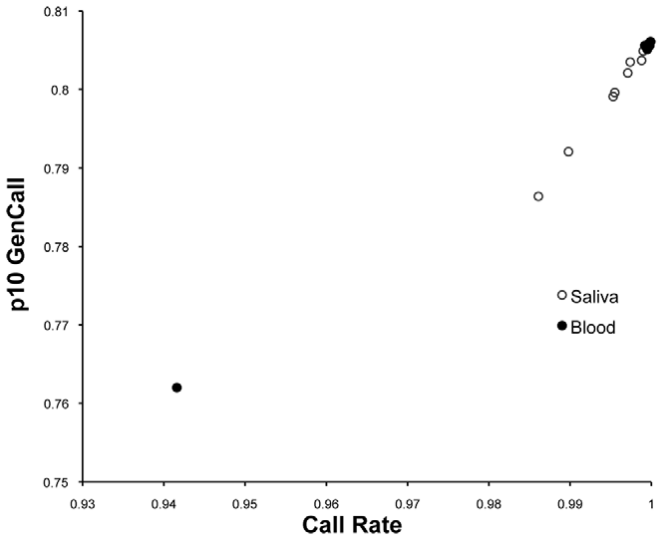
Plot of 10^th^ percentile of range of GenCall score versus call rate after quality control for saliva- and blood-extracted DNA samples. Sample 10% GenCall score is plotted against sample call rate as a means of visualizing overall sample performance. Each sample is represented by one data point, with saliva samples represented by open circles and comparison blood samples as filled circles. The poorly performing blood sample is in the lower left quadrant. (Note axes do not start at the origin.) P10 GenCall–10% GenCall score. See Figure S1 for sample performance as compared to total blood samples genotyped.

Illumina's Infinium Canine SNP20 genotyping array was developed by Illumina to survey the canine genome at sufficient coverage for use in GWAS as suggested by Lindblad-Toh *et al.* (2005). The array contains 22,362 SNPs with a median of 565 markers per chromosome (mean 573.4, maximum 1146, minimum 267). The average intermarker spacing is 103.6 kb, with median intermarker spacing of 67.8 kb. Several very large gaps inflate this mean, with the largest gap at 5.6 Mb (on the X chromosome). There are three, four and 27 gaps >3 Mb, >2 Mb and >1 Mb in size, respectively, and 292 SNPs with gaps >500 kb. The average call rate for the 22,362 SNPs surveyed by the Infinium CanineSNP20 array before quality control (QC) of SNP data was 99.2% for saliva samples (n = 11) and 98.5% for comparison blood samples (n = 5; Table 2). This compared to an average call rate of 99.4% for all blood samples genotyped on this platform by our group (n = 192, data not shown). The mean genotyping statistics for the five comparison blood samples included in this report are lower than the overall average we saw in our total samples because of one poor-performing sample (see Table S1). When this poorly performing sample was removed, the mean call rate was 99.6% (Table 2). After QC, 20,753 SNPs remained, with average call rates of 99.6% for saliva samples and 98.8% for all comparison blood samples (Table 2). Another useful metric for evaluating sample quality and performance is the Illumina GenCall score (GenCall Version 6.3.0), which is calculated for each genotype. GenCall scores range from 0 to 1, with smaller values representing data points that fall further from the center of the genotype call cluster with which the sample is associated. Genotypes with a GenCall score≤0.15 received no call. Post-QC, average Illumina 10% GenCall scores—the 10^th^ percentile (p10) of the range of GenCall scores across all genotypes called for the individual—were 0.801 and 0.797 for saliva and comparison blood, respectively (Table 2; the average for all blood samples genotyped by our group on this platform was 0.803, data not shown). Plotting call rate versus p10 GenCall scores demonstrated that all saliva samples performed equally well as comparison blood samples after QC (Figure 1; Figures S1 and S2). Mean genotyping statistics excluding the performance outlier are also provided in Table 2, and genotyping statistics after QC for each sample are given in Table S1.

**Table 2:** Genotyping statistics for saliva- and blood-extracted DNA samples before and after marker quality control (QC).

			Mean (range)	
Source	n	# SNPs	Call Rate (%)	p10 GenCall
***Pre-QC***				
Saliva	11	22,362	99.2 (98.5–99.6)	0.791 (0.781–0.797)
Blood	5	22,362	98.5 (94.2–99.6)	0.787 (0.748–0.798)
Blood (no outlier)	4	22,362	99.6 (99.5–99.6)	0.797 (0.796–0.798)
***Post-QC***				
Saliva	11	20,753	99.6 (98.6–100)	0.801 (0.786–0.806)
Blood	5	20,753	98.8 (94.2–100)	0.797 (0.762–0.806)
Blood (no outlier)	4	20,753	100 (99.9–100)	0.806 (0.805–0.806)

Mean values plus ranges for the call rate and 10^th^ percentile of the range of the GenCall scores for each sample type before and after marker QC. Source–tissue source of DNA extraction; n–number of dogs; # SNPs–number of total markers used for calculating statistics across samples; p10 GenCall–10^th^ percentile of GenCall score range; pre-QC–raw data before marker quality control; post-QC–cleaned marker set after quality control (see *Methods* section for marker exclusion criteria).

**Figure 2:**
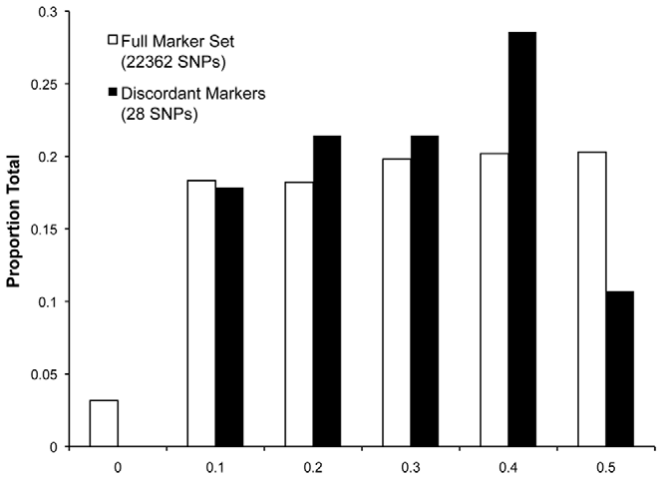
Allele distributions for discordant markers compared to full marker set. Histogram of allele frequencies for the full marker set (open bars) and discordant SNPs (solid bars). Discordant markers appear to trend towards larger allele frequencies. X-axis –allele frequency upper bound for bin; Y-axis–proportion of total (respective) marker set.

Of the 11 saliva samples genotyped, five dogs were also represented by blood samples. For four of the replicate samples, mean concordance of called genotypes in both samples (saliva and blood) was 99.98% (Table 1). One replicate sample was dropped from our analysis due to low concordance that suggested within-breed sample mixing (Table S1). We examined the characteristics of the SNPs responsible for sample discordance in the remaining four samples to see if particular marker characteristics may predict discordance. However, we found that only one out of 28 discordantly-called SNPs had >1 discordant call, whereas the majority were discordant singletons (Table 3). Binning markers by minor allele frequency (MAF) suggested a trend towards higher frequencies in discordantly-called SNPs (Figure 2), though the mean MAF for the 28 discordant SNPs was very similar to that of the entire marker set (Table 3). It also appears that the discordant SNPs had lower performance than the full marker set; however, the averages between the two sets were not markedly different (Table 3).

**Table 3:** Mean genotyping statistics for discordantly-called markers compared to full marker set.

		Mean Call Statistics				
SNP set	# SNPs	Call Rate (%)	Rep Errors	MAF	GenTrain Score	p10 GC
Full Marker Set	22362	99.4	–	0.248	0.854	0.871
Discordant SNPs	28	98.7	1.036	0.241	–	0.763
>1 Discordant calls	1	96.9	2	0.449	–	0.541

Mean statistics are provided for the full marker set (before QC) as well as for SNPs whose genotypes were called discordantly between saliva versus blood replicate samples. # SNPs–number of total markers used for calculating statistics across samples; Rep Errors–number of replicate errors (discordant genotype calls); MAF–minor allele frequency; p10 GC–10^th^ percentile of GenCall score range.

### CNVs

**Figure 3:**
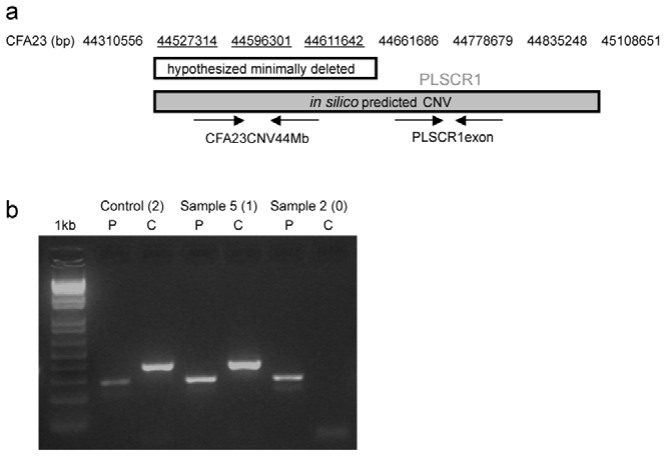
Molecular evaluation of putative CNV region on chromosome 23. (a) Two amplicons within the *in silico* predicted copy variable region on chromosome 23 were evaluated for presence/absence in saliva samples that had called copy loss. Base position (bp) on chromosome 23 is given at top of diagram (not to scale). PLSCR1exon-amplicon is an exonic region of the PLSCR1 gene; CFA23CNV44Mb-amplicon is a predicted conserved region in the middle of a hypothesized minimally deleted block based on no-call genotypes for three SNPs spanning this region (indicated by underlined base positions). (b) PCR amplicons visualized by UV on 2% agarose gel with 1 kb DNA ladder for reference. Predicted size is 293 and 389 bases for the PLSCR1exon (“P”) and CFA23CNV44Mb (“C”) amplicons, respectively. Sample identities are provided with predicted copies present in parenthesis; Control-Lab Control blood sample predicted to have no loss (i.e. 2 copies present).

Copy number variation can be readily evaluated with SNP data within the GenomeStudio software package. CNVs called *in silico* were evaluated in all genotyped samples and those appearing to specifically include a subset of the saliva-extracted samples were further assessed for validity via manual inspection of genotype data. A region on chromosome 23 had copy number losses predicted for Sample 2 (homozygous loss) and Sample 5 (heterozygous loss), as well as predicted homozygous loss in three other Bearded Collie blood samples (data not shown). To validate these calls, we investigated this region via direct PCR of genomic samples for two amplicons located within the putative deletion region: PLSCR1exon amplicon designed to span the 8th exon of the PLSCR1 gene, and CFA23CNV44Mb amplicon designed to span a predicted conserved region (annotated in the UCSC Genome Browser) in the middle of a hypothesized minimally deleted region based on no-call genotypes in the three samples predicted to have homozygous deletions (Figure 3a). PCR results confirmed deletion of the hypothesized minimally deleted region in all samples with homozygous deletion calls but presence of the PLSCR1exon region as expected from present genotype calls in homozygous loss samples (Figure 3b).

## Discussion

Our results demonstrate that saliva collection from dogs is facile, convenient, and yields large amounts of high-quality DNA that provide excellent performance on high-throughput whole genome arrays. Overall, the DNA yield our group obtained was similar to that found in a previous study examining human saliva specimens [13]. Our mean yield was higher than another research group's (Mitsouras et al., 2009) but lower than the reported yield by the kit manufacturer for canine samples, although it should be noted that Iwasiow *et al.* [15] report corrected ratios that were adjusted for presence of turbid material that absorb at 320 nm, a step that we elected not to perform. The DNA purity we obtained (as measured by 260/280 ratios) was, however, similar to reported values for both human and canine saliva samples (Table S1). Our results also suggest that the extraction method used for saliva samples is important, and that subtle differences in extraction protocols may produce differences in DNA purity and/or introduce contaminants, though in our case this did not appear to alter array performance. Whether or not NaCl was used in extractions appeared to produce slight differences in yield, but did not appear to alter DNA purity or level of contamination as measured by 260/280 or 260/230 ratios, respectively (Table S1). Other aspects of quality to be explored in the future include measuring levels of contaminating RNA and microbial DNA load, determining if DNA is of high molecular weight, and investigating long-term stability post-extraction.

High genotype concordance between blood and saliva samples suggests a high level of fidelity for genotype controls from saliva-extracted DNA, and were similar to replicated blood-extracted samples from the same individual (data not shown). However, because DNA from different tissues may produce source-specific profiles with regard to probe fluorescence (Figure S2)—which may ultimately affect genotype calling—it is prudent that samples for association studies have balanced representation of cases and controls from each DNA source to reduce spurious associations due strictly to tissue type, sample provenance, and genotyping batch effects [16]. Excluding the outlier sample (which was suspected of sample mixing) and examining the SNPs that were called discordantly between highly concordant replicates, we found only one marker that was called discordant >1 time. This suggests the discordance is random, and that saliva does not lead to differential discordance when compared to blood.

Because they provide high-fidelity SNP genotypes, it appears that saliva-extracted samples can also be used for successful CNV calling *in silico*. Calls are made for putative CNVs and regions of homozygosity based on genotypes across multiple markers. It thus follows that the size of putative copy variable regions relies on the density of the SNP data, and that the size of a reported CNV may be artifactually large due to the requirements of the calling algorithm. Because of the large inter-SNP distances in our data set, direct assessment of genotype calls was therefore also used to hypothesize a *minimally* deleted region in our samples (where no-calls suggest absence of region), which we verified by direct amplification of genomic DNA. Our results demonstrate that saliva samples can also be used reliably in copy variation analysis, although similar requirements for case-control tissue sample consistency still apply.

We found one saliva sample that demonstrated low concordance (82.6%) with its replicate blood sample. This was likely due to switching samples of dogs within the same breed, as pairwise concordance between known but different dogs of the same breed was similar to that seen in our low-concordance sample, whereas concordance between known dogs of different breeds was much lower (Table S2). Further, our calculations of concordance between known related versus known unrelated dogs suggested that sample switching likely occurred between related dogs. Our analysis also suggested the switching was specifically in the saliva sample, as concordance between the saliva sample and the dog's sire was lower than that of the blood sample and sire. The concordance we saw between the saliva versus blood samples was similar to that of distantly-related dogs, which suggests the sample switching could have resulted from mislabeling or sampling the wrong dog from a household with multiple related dogs (Table S2). Additionally, chimeric samples due to dogs licking each other or sharing water bowls could produce heterogeneous genotyping results and warrants further investigation, although this would likely generate heterozygosity outliers.

One caveat of this work is that the blood samples were genotyped on a separate run several months earlier than the saliva samples, which could introduce artifacts when comparing genotypes and statistics for samples representing the same individual. However, it is more likely that these artifacts would introduce inconsistencies between duplicated samples; this would result in an underestimation of the total concordance seen between duplicates in our study. As our concordances are already greater than 99% (excluding the suspected wrong sample pairing), this suggests that even higher fidelity in genotype calls between blood- versus saliva-extracted DNA samples may be possible if all samples are run in the same genotyping batch. The similarity of clustering data also suggests that samples of diverse provenance can be clustered together using Infinium data. We have recently observed high concordance rates (99.9999%) between blood and saliva replicate pairs on the next generation Illumina canine array with 170,403 QC-filtered SNPs, with a mean call rate for saliva samples of 99.78% (n = 3, data not shown).

One limitation to this study is the ascertainment bias introduced by our study design. Because we required prior written assent from owners to participate in saliva sample collection prior to kits being sent out, it is likely that our return rates are overestimates of the population at large. However, because saliva collection is so simple and non-invasive, it is probable that return rates would be quite significant, and likely higher than the rate of blood sample collection. Another limitation of this study is the small number of duplicated samples. This limitation highlights the need for replication with larger numbers of dogs from different breeds (large and small), and on different genotyping platforms by other groups for further validation of the performance of saliva-extracted DNA for high-throughput assays.

In summary, we demonstrate for the first time that saliva sample collection in dogs is a noninvasive means of obtaining high quality DNA for successful use with genome-wide array genotyping, with little danger of loss of information due to the source of data. The dual conveniences of owner sampling in the home and ease of shipping provide alternative means of obtaining samples from rural locales or foreign countries where collection of blood samples may be difficult or impossible. Additionally, ease of sampling allows for collection of large numbers of samples with minimal investment of time and manpower, creating potential for collecting an entire study cohort at a small number of targeted sampling events. Finally, the non-invasive nature of saliva collection makes it particularly appealing when studying dogs whose conditions may otherwise prevent blood collection, such as high levels of anxiety or repeated use of veins for other medical purposes related to disease status. In sum, these factors will lead to increased sample return rates which will increase study sizes and ultimately enhance the ability for geneticists to detect novel genetic loci underlying disease and behavioral traits in a GWAS framework.

## Supporting Information

Table S1 Individual saliva and comparison blood sample statistics for DNA extraction and genotyping compared to published data for human saliva and blood, plus manufacturer's report. Individual statistics are given for each saliva and comparison blood sample for DNA concentration (ng/ul), DNA purity (260/280), contamination (260/230), post-QC genotype call rate and post-QC p10 GenCall score (p10 GC). Saliva vs. blood sample concordance rates are also given for every individual represented by both tissue types. Mean values as reported in the main text are provided, as well as the published values for human saliva and blood samples as reported by Hansen et al. and dog saliva statistics as reported by researchers from the manufacturer of the Oragene ANIMAL collection kit, DNA Genotek (Iwasiow 2009).
(0.27 MB DOC)

Table S2 Replicate statistics. Sample identification, tissue source, breed, geographic origin (US vs. foreign) and gender are given for samples (A versus B) that were compared for replicate (concordance) statistics. Sample 2 is suspected to be a switched sample, and demonstrates similar concordance rates as distantly-related dogs of the same breed. Samples 11 & 12 are dogs from a geographically distinct population (Yokoyama et al., in preparation). BEC = Bearded Collie; BOC = Border Collie. # Correct-total concordant genotype calls; # Errors-total discordant genotype calls; Total-total number of markers with genotype calls in both samples; Rep Freq-replicate frequency (concordance rate); Relation-unrelated refers to dogs that share no grandparents.
(0.29 MB DOC)

Figure S1 Plot of p10 GenCall score versus call rate after quality control for saliva-and blood-extracted DNA samples compared to full set of genotyped samples. Sample 10% GenCall score is plotted against sample call rate as a means of visualizing overall sample performance. Each sample is represented by one data point, with saliva samples represented by open circles, comparison blood samples as filled circles and remaining blood samples also genotyped by our group as grey circles (n = 192-Yokoyama et al., in preparation). Overall, saliva samples performed in the same range as all blood samples genotyped. (Please note axes do not start at the origin.) P10 GenCall-10% GenCall score.
(0.14 MB DOC)

Figure S2 Cluster plots for select SNPs. Saliva- versus blood-extracted DNA samples (by columns) are highlighted in cluster plots of genotyped samples (n = 192-Yokoyama et al., in preparation) from GenomeStudio.
(0.28 MB DOC)
