# Supplementary material for: Array-Based Whole-Genome Survey of Dog Saliva DNA Yields High Quality SNP Data
Source: PLoS One. 2010 May 25;5(5):e10809. doi: 10.1371/journal.pone.0010809 (PMC2876042; doi:10.1371/journal.pone.0010809)
Supplement: Table S1 — Individual saliva and comparison blood sample statistics for DNA extraction and genotyping compared to published data for human saliva and blood, plus manufacturer's report. Individual statistics are given for each saliva and comparison blood sample for DNA concentration (ng/ul), DNA purity (260/280), contamination (260/230), post-QC genotype call rate and post-QC p10 GenCall score (p10 GC). Saliva vs. blood sample concordance rates are also given for every individual represented by both tissue types. Mean values as reported in the main text are provided, as well as the published values for human saliva and blood samples as reported by Hansen et al. and dog saliva statistics as reported by researchers from the manufacturer of the Oragene ANIMAL collection kit, DNA Genotek (Iwasiow 2009). (0.27 MB DOC) [file pone.0010809.s001.doc]

|  | **Saliva Samples** | | | | | **Blood Samples** | | | | |
| --- | --- | --- | --- | --- | --- | --- | --- | --- | --- | --- |
|  | **ng/ul** | **260/280** | **260/230** | **Call Rate** | **p10 GC** | **ng/ul** | **260/280** | **260/230** | **Call Rate** | **p10 GC** |
| Laboratory Control | 46.91 | 1.79 | 0.44 | 0.999 | 0.805 | 317.00 | 2.35 | 0.47 | 0.999 | 0.806 |
| Sample 1^a^ | 71.21 | 1.50 | 0.34 | 0.990 | 0.792 | 521.22 | 1.86 | 1.93 | 0.942 | 0.762 |
| Sample 2 | 127.05 | 1.75 | 0.63 | 0.999 | 0.805 | 345.50 | 1.88 | 1.89 | 1.000 | 0.805 |
| Sample 3 | 171.16 | 1.80 | 0.76 | 0.997 | 0.804 | 369.31 | 1.86 | 1.95 | 1.000 | 0.806 |
| Sample 4 | 212.43 | 1.84 | 0.79 | 0.995 | 0.799 | 369.05 | 1.84 | 1.80 | 1.000 | 0.806 |
| Sample 5 | 83.93 | 1.56 | 0.37 | 1.000 | 0.806 | *NA* | *NA* | *NA* | *NA* | *NA* |
| Sample 6 | 100.38 | 1.80 | 0.61 | 0.997 | 0.802 | *NA* | *NA* | *NA* | *NA* | *NA* |
| Sample 7 | 92.71 | 1.86 | 0.71 | 0.996 | 0.800 | *NA* | *NA* | *NA* | *NA* | *NA* |
| Sample 8 | 146.41 | 1.58 | 0.44 | 0.986 | 0.786 | *NA* | *NA* | *NA* | *NA* | *NA* |
| Sample 9 | 161.95 | 1.39 | 0.33 | 0.999 | 0.804 | *NA* | *NA* | *NA* | *NA* | *NA* |
| Sample 10 | 165.97 | 1.45 | 0.39 | 1.000 | 0.806 | *NA* | *NA* | *NA* | *NA* | *NA* |
| **Mean** | **125.46** | **1.67** | **0.53** | **0.996** | **0.801** | **384.42** | **1.96** | **1.61** | **0.988** | **0.797** |
| **Mean (no outlier^a^)** | **-** | **-** | **-** | **-** | **-** | **-** | **-** | **-** | **1.000** | **0.806** |
| Hansen *et al.* (2007 - humans) | 108.00 | 1.63 | 0.80 | *NA* | *NA* | 56.80 | 1.79 | 1.44 | *NA* | *NA* |
| Iwasiow *et al.* (2009 - dogs) | 232.00 | 1.60 | *NA* | *NA* | *NA* | *NA* | *NA* | *NA* | *NA* | *NA* |
| *^a^Sample 1 removed as blood sample genotyping performance outlier* | | | | | | |  |  |  |  |
