# Supplementary figures and images for: Array-Based Whole-Genome Survey of Dog Saliva DNA Yields High Quality SNP Data
Source: PLoS One. 2010 May 25;5(5):e10809. doi: 10.1371/journal.pone.0010809 (PMC2876042; doi:10.1371/journal.pone.0010809)

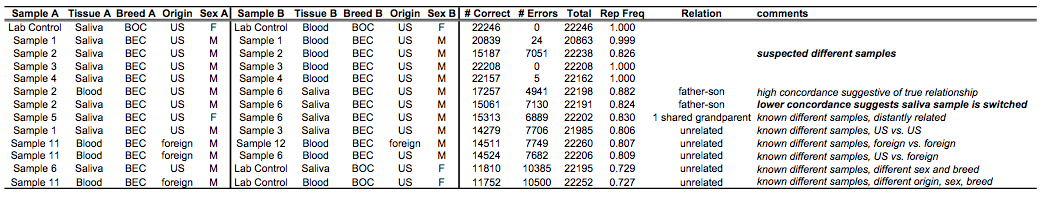

Supplement: Table S2 — Replicate statistics. Sample identification, tissue source, breed, geographic origin (US vs. foreign) and gender are given for samples (A versus B) that were compared for replicate (concordance) statistics. Sample 2 is suspected to be a switched sample, and demonstrates similar concordance rates as distantly-related dogs of the same breed. Samples 11 & 12 are dogs from a geographically distinct population (Yokoyama et al., in preparation). BEC = Bearded Collie; BOC = Border Collie. # Correct-total concordant genotype calls; # Errors-total discordant genotype calls; Total-total number of markers with genotype calls in both samples; Rep Freq-replicate frequency (concordance rate); Relation-unrelated refers to dogs that share no grandparents. (0.29 MB DOC) [file pone.0010809.s002.doc]

**
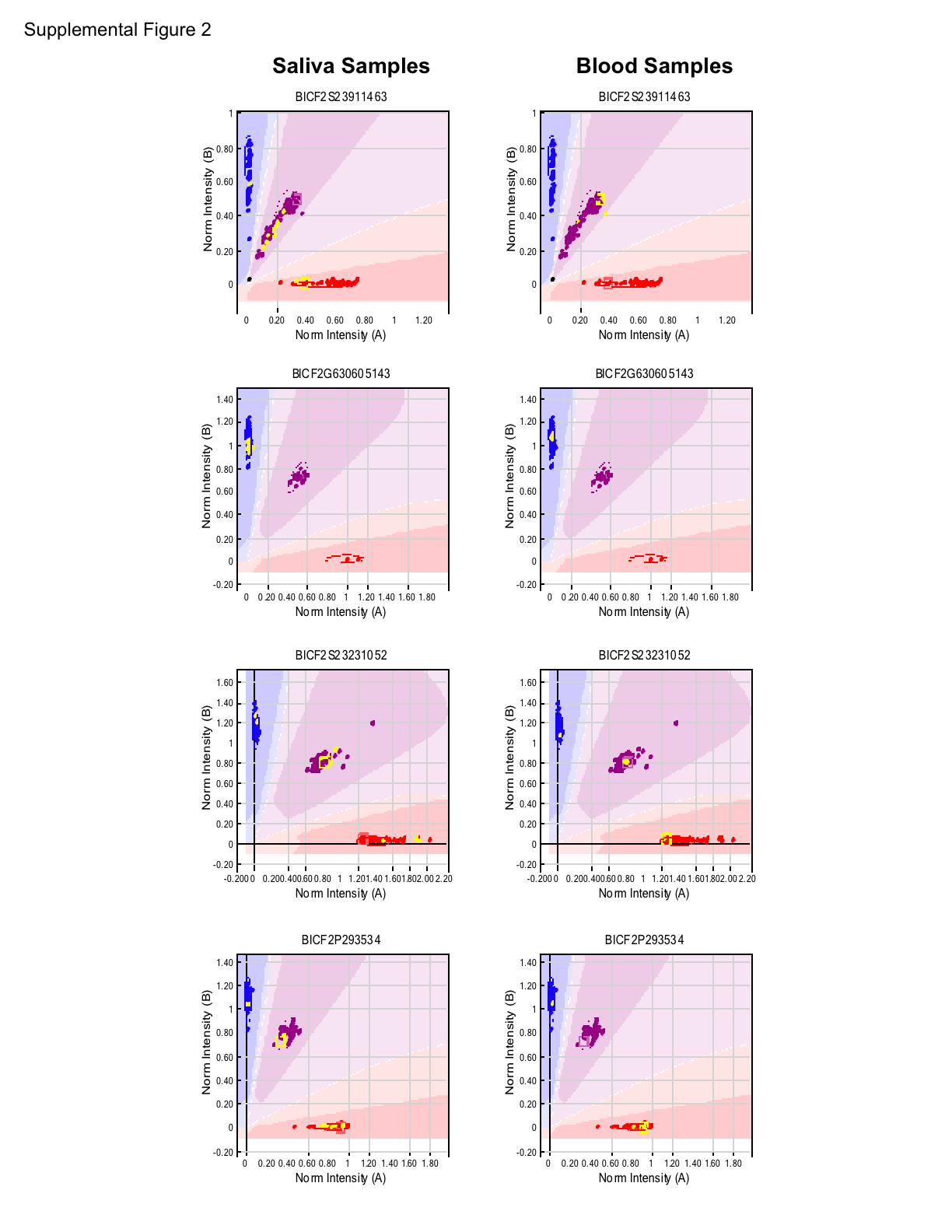
**

Supplement: Figure S2 — Cluster plots for select SNPs. Saliva- versus blood-extracted DNA samples (by columns) are highlighted in cluster plots of genotyped samples (n = 192-Yokoyama et al., in preparation) from GenomeStudio. (0.28 MB DOC) [file pone.0010809.s004.doc]
