# Supplementary material for: Array-Based Whole-Genome Survey of Dog Saliva DNA Yields High Quality SNP Data
Source: PLoS One. 2010 May 25;5(5):e10809. doi: 10.1371/journal.pone.0010809 (PMC2876042; doi:10.1371/journal.pone.0010809)
Supplement: Supplementary file 3 [file pone.0010809.s003.doc]

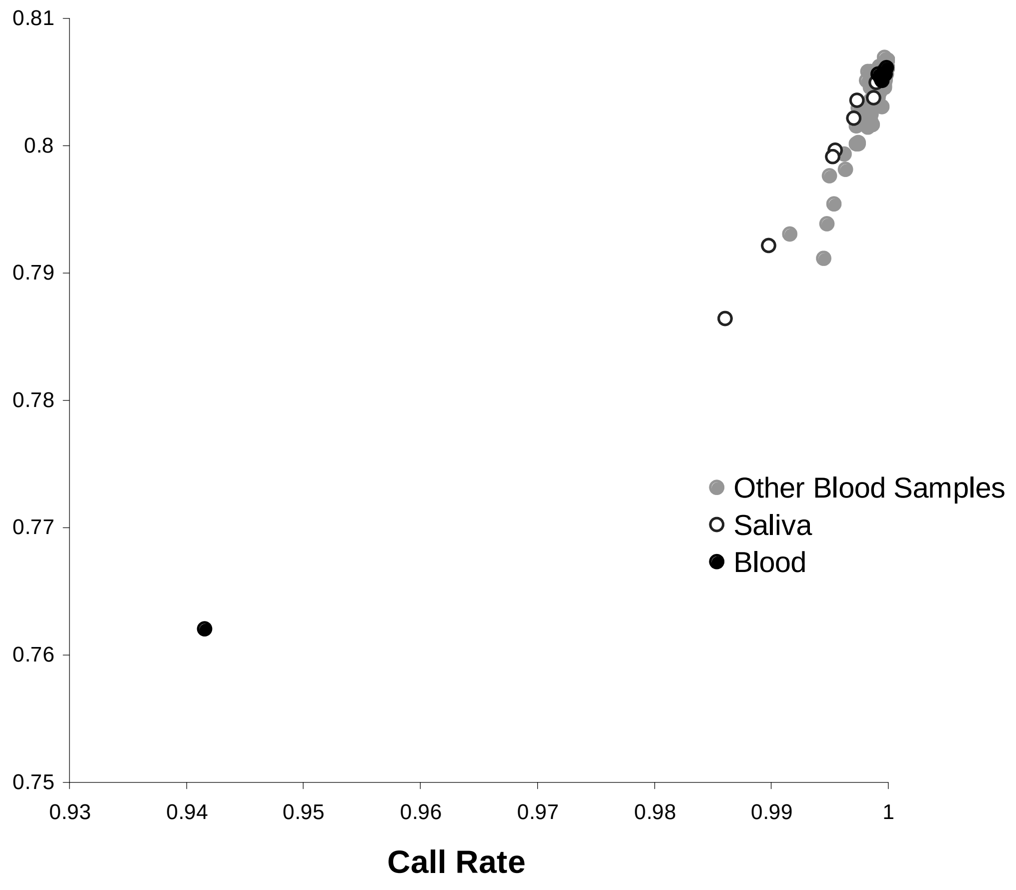
0.81
0.8
0.79
0.78
0.77
0.76
0.75
0.93
0.94
0.95
0.96
0.97
0.98
0.99
1
Call Rate
Other Blood Samples
Saliva
Blood
